# Timing and outcome of bystanders treatment in patients with subarachnoid hemorrhage associated with multiple aneurysms

**DOI:** 10.1007/s10143-022-01799-z

**Published:** 2022-05-03

**Authors:** Carmelo Lucio Sturiale, Anna Maria Auricchio, Vito Stifano, Rosario Maugeri, Alessio Albanese

**Affiliations:** 1 Department of Neurosurgery, Fondazione Policlinico Universitario A. Gemelli IRCCS, Università Cattolica del Sacro Cuore, L.go A. Gemelli, 8 - 00168 Rome, Italy; 2 Neurosurgical Clinic, AOUP Paolo Giaccone, Department of Biomedicine Neurosciences and Advanced Diagnostics, School of Medicine, University of Palermo, Palermo, Italy

**Keywords:** Multiple intracranial aneurysm, Subarachnoid hemorrhage, Bystander aneurysms, Timing, PHASES

## Abstract

In case of subarachnoid hemorrhage (SAH) associated with multiple intracranial aneurysms (MIAs), the main goal of acute treatment is securing the source of bleeding (index aneurysm). Indications and timing of bystanders treatment are instead still debated as the risk of new SAHs in patients harboring MIAs is not yet established. However, even if technically feasible, a simultaneous management of all aneurysms remains questionable, especially for safety issues. We retrospectively reviewed our last 5-year experience with SAH patients harboring MIAs entered in a clinic-radiological monitoring for bystanders follow-up in order to evaluate the occurrence of morphological changes, bleeding events, and safety and efficacy of a delayed treatment. We included 39 patients with mean age of 59.5 ± 12.2 years who survived a SAH. Among them, 14 underwent treatment, whereas 25 continued follow-up. The mean time between index and bystanders treatment was 14.3 ± 19.2 months. Patients undergoing bystanders treatment were mainly female and in general younger than patients undergoing observation. No cases of growth or bleeding were observed among bystanders within the two groups during the follow-up, which was longer than 1 year for the intervention group, and almost 40 months for the observation group. No major complications and mRS modifications were observed after bystanders treatment. Our data seem to suggest that within the short follow-up, intervention and observation seem to be likewise safe for bystander aneurysms, showing at the same time that a delayed management presents a similar risk profile of treating unruptured aneurysms in patients with no previous history of SAH.

## Introduction

In case of subarachnoid hemorrhage (SAH) associated with detection of multiple intracranial aneurysms (MIAs), the main goal of the first diagnostic round is to detect the source of bleeding (index aneurysm). Then, the principal aim of the treatment is securing the ruptured aneurysm to prevent rebleeding, which may represent a life-threatening condition [1].

On the other hand, indications and timing for treating the concomitantly detected unruptured aneurysms (bystanders) are still debated, and clear recommendations are not yet available. In fact, even when technically feasible, the benefit to treat index and bystanders at the same time remains questionable, especially for safety issues.

In this paper, we collected our recent experience with SAH patients harboring MIAs to review our attitude in bystanders treatment and evaluated (a) indications; (b) risk of a delayed treatment; and (c) complications and outcome of a second surgical or interventional procedure at distance from a previous SAH.

## Materials and methods

We retrospectively reviewed all consecutive cases of patients admitted to our department from January 2016 to December 2020 for aneurysmal SAH. After a complete neuroimaging screening, only patients harboring MIAs at the urgent head CT angiography (CTA) were included. All these patients also had one or more unruptured aneurysms for which they were included in a follow-up program after the treatment of the index aneurysm.

Clinical presentation after SAH was scored according to the Hunt-Hess scale, while SAH pattern was classified according to the modified Fisher grading. Clinical outcome was instead scored according to modified Rankin scale and assessed at three time points: (a) at discharge, (b) after the rehabilitation program and before the second admission for bystanders treatment, and (c) at last follow-up.

At the time of the first SAH, all patients underwent a baseline head CT scan using a 64-slice multi-detector device (Philips Healthcare™) and a CTA with a field of view of 160 mm and a slice thickness of 1 mm reconstructed at 0.5 mm, resulting in a voxel size of 0.3 × 0.3 × 0.5 mm. We usually considered three main parameters for the identification of the index (ruptured) aneurysm: (1) blood distribution pattern; (2) morphometric characteristics of the aneurysm; (3) neurological signs. If the ruptured aneurysm was not identified after the first round of neuroimaging, patients underwent a diagnostic DSA with 3D rotational angiograms, followed by a multidisciplinary discussion for a final decision. DSA was also indicated in case of consensus for an endovascular approach.

We basically agreed about the policy of “endovascular first” for all ruptured aneurysms amenable for a simple coiling stand-alone and in general for most of posterior circulation aneurysms. A policy of “surgery first” was usually adopted in case of SAH associated with ICH requiring evacuation; in all cases needing of a complex endovascular approach with coiling plus adjuncts, which often also required acute antiplatelet administration; for almost all cases of MCA aneurysms. Older age and comorbidities were usually the most important variables influencing the decision-making process.

Then, patients were addressed to endovascular or microsurgical treatment within 24 h from initial presentation, usually to treat the index aneurysm. For a minority of patients whose index aneurysms were not recognized, urgent treatment was addressed to secure multiple aneurysms at the same time.

For the aim of the present study, we selected only patients whose bystanders treatment was postponed. For these patients, we reviewed all clinical and neuroradiological data collected during the follow-up period.

We investigated all demographical and clinical aspects influencing the decision to treat or observe the bystanders, including some major risk factors such as smoke habits and hypertension.

We also investigated the neuroimaging modality (CTA/MRA/DSA) used for the follow-up and assessed bystanders topography, morphology, size, and the other angioarchitectural features in order to detect any morphological change occurring over time, including bystander rupture during the follow-up.

Then, we retrospectively calculated the PHASES score for each bystander to verify its agreement or disagreement with the institutional neurovascular board consensus.

Finally, we divided patients into two groups according to the decision to treat or follow-up and compared all the patients’ clinical and neuroradiological characteristics between the two groups.

Quantitative variables were expressed as mean ± standard deviation and the Student *t*-test was used to compare their means. The Fisher exact test (two-sided) was used to compare the categorical variables with the outcome.

Statistical analysis was done using Microsoft Excel v.16 (Microsoft, Redmond, WA, USA) and R software (version 4.0).

## Results

Between January 2016 and December 2020, 265 patients presented at our emergency department with aneurysmal SAH. Among them, 62 patients (9 males and 53 females, mean age 60.0 ± 11.8 years) showed multiple aneurysms (overall 161).

At the end of the first- and second-level diagnostic work-up, the source of bleeding (index aneurysm) was identified with certainty in 56 out of 62 cases (90.3%).

In 6 patients (9.7%), we instead failed the identification of the bleeding source and then proceeded to a multiple clipping or coiling in the same intervention.

The remaining 56 patients undergoing urgent treatment of the index aneurysms, also harbored further 81 unruptured bystanders, which were included in a follow-up program.

Among them, 17 (30.3%) had a clinical onset characterized by a high (12 pts HH5; 1 with HH4) or intermediate Hunt-Hess grade (2 with HH3; 1 with HH2) and died within 1 month for a direct consequence or complications of the SAH. Along the subsequent clinical and neuroradiological follow-up, no cases of new SAH from bystander rupture were observed.

We included in our study the 39 patients who survived the SAH (mean age 59.5 ± 12.2 years; male to female ratio 8:31) and entered a follow-up program (mean follow-up time 36.26 ± 19.17 months) including a long-term rehabilitation plan, and a periodical clinic-radiological reassessment.

Demographic and clinical characteristics of the studied population are reported in Table 1.

**Table 1:** Demographic and clinical characteristics of the studied population

		**Treated patients** ***n* = 14 (%)**	**Observed patients** ***n* = 25 (%)**	***p*-value**
**Age ± SD**		51.3 ± 7.4	58.2 ± 11.8	**0.05**
**Female sex**		14 (100)	17 (68)	**0.03**
**No. of aneurysms**		15	37	-
**Bystanders topography in respect to index**	**Ant vs post circulation**	13 vs 2	29 vs 8	0.70
	**Ipsilateral vs contralateral side**	14 vs 1	23 vs 14	**0.04**
**mRS at discharge after SAH**	**0**	4 (28.6)	6 (24)	1.00
	**1**	4 (28.6)	3 (12)	0.22
	**2**	2 (14.3)	4 (16)	1.00
	**3**	3 (21.4)	9 (36)	0.47
	**4**	1 (7.1)	2 (8)	1.00
	**5**	0 (0)	1 (4)	1.00
**Hypertension**		11 (78.6)	23 (92)	0.32
**Smoke habits**		7 (50)	9 (36)	0.50
**Radiological follow-up assessment**	**CTA**	5 (35.7)	13 (52)	0.50
	**DSA**	9 (64.3)	4 (16)	** < 0.01**
	**MRA**	0 (0)	8 (32)	**0.03**

Based on the institutional neurovascular board consensus, 14 among these 39 patients, harboring overall 15 unruptured aneurysms, underwent treatment, whereas 25 continued the bystanders observation without intervention.

“Size larger than 4 mm, irregular shape, presence of blebs, topography and in general the possibility of a simple coiling stand-alone as well as some main risk factors as hypertension, long-time smoke habit, familiarity for SAH and patients preference were the key-factors guiding the neurovascular board consensus”.

The mean time between index and bystanders treatment was 14.3 ± 19.2 months and this time span was mainly influenced by patients’ clinical condition.

Patients harboring bystander aneurysms that were considered at higher risk of rupture underwent a shorter neuroradiological follow-up, more often with DSA or CTA. MRA was mainly reserved for those harboring smaller bystanders included in a long-term follow-up program (Table 1).

Patients who underwent bystander aneurysm treatment were predominantly female (*p* = 0.03) and usually younger than those undergoing only observation (51.3 ± 7.4 vs 58.2 ± 11.8; *p* = 0.05). The two groups of patients did not appear instead significantly different in terms of incidence of hypertension and persistent smoke habit.

The aneurysm angioarchitectural features of the two groups are listed in Table 2.

**Table 2:** Aneurysm characteristics of the included patients

		**Treated aneurysms** ***n* = 15 (%)**	**Observed aneurysms** ***n* = 37 (%)**	***p*-value**
**Topography**	**ACA**	0 (0)	7 (18.9)	0.09
	**MCA**	5 (33.3)	9 (24.3)	0.51
	**ICA**	8 (53.3)	12 (32.4)	0.21
	**ACom**	0 (0)	2 (5.4)	1.00
	**PCA**	0 (0)	1 (2.7)	1.00
	**PICA**	2 (13.3)	1 (2.7)	0.19
	**BA**	0 (0)	1 (2.7)	1.00
	**SCA**	0 (0)	3 (8.1)	0.54
	**VA**	0 (0)	1 (2.7)	1.00
**Saccular morphology (versus fusiform)**		15 (100)	36 (97.3)	1.00
**Mean size ± SD (mm)**		5.9 ± 4.2	2.5 ± 1.5	** < 0.01**
**Mean AR ± SD**		2.6 ± 3.7	1.3 ± 0.7	**0.04**
**Presence of blebs**		1 (6.7)	0 (0)	0.30
**Irregular shape**		5 (33.3)	1 (2.7)	** < 0.01**
**Surgical treatment (vs endovascular)**		7 (46.7)	NA	-
**SAH from bystander aneurysms**		0 (0)	0 (0)	1.00
**Bystander aneurysm growth at FU**		0 (0)	0 (0)	1.00
**PHASES** **(5-year risk of rupture in %)**	**0–1**	0 (0)	0 (0)	1.00
	**2–4**	5 (33.3)	12 (32.4)	1.00
	**5–9**	10 (66.6)	24 (64.9)	1.00
	** > 9**	0 (0)	1 (2.7)	1.00
**Aneurysm remnants after treatment**		3 (20)	NA	-

**Legend**: *AR*, aspect ratio; *FU*, follow-up; *NA*, not applicable; *SAH*, subarachnoid hemorrhage; *SD*, standard deviation

The 15 bystanders which were treated originated from ICA in 8 cases (53.3%), from MCA in 5 cases (33.3%), and from PICA (13.3%), but there was not a significant difference in their topographical distribution compared with those undergoing observation. The two groups were instead significantly different in terms of mean size, which appeared roughly double for aneurysms of the treatment group (5.9 ± 4.2 versus 2.5 ± 1.5; *p* < 0.01) as well as the aspect ratio (2.6 ± 3.7 versus 1.3 ± 0.7; *p* = 0.04). Most of the treated aneurysms also showed an irregular shape (33.3% versus 2.7%, *p* < 0.01), but with no major incidence of blebs on their surface. On the other hand, none of the PHASES score categories appeared significantly different between the two groups.

The early mRS assessment after the SAH did not appear a limiting factor for future bystanders treatment as it generally improved over time after the rehabilitation program. In fact, more than 90% of the patients who underwent a secondary bystanders treatment had a good quality of life (mRS 0–1) after the completion of the rehabilitation program. Moreover, none among them showed a worsening in their quality of life at last clinical follow-up that was on average more than 1 year after second surgery, and more than 2.5 years from the first one (Table 3). Five patients within the observed group were lost at follow-up as they died. Two of them had a concomitant oncologic disorder, while the other three had systemic respiratory infectious complications as they hesitated in a severe clinical condition after the first SAH (Table 3).

**Table 3:** Clinical outcome and complications of included patients

		**Treated patients** ***n* = 14 (%)**	**Observed patients** ***n* = 25 (%)**	***p*-value**
**mRS before the bystanders treatment**	0	12 (85.7)	9 (36)	** < 0.01**
	1	1 (7.1)	7 (28)	0.21
	2	0 (0)	3 (12)	0.54
	3	1 (7.1)	4 (16)	0.63
	4	0 (0)	1 (4)	1.00
	5	0 (0)	1 (4)	1.00
	6	NA	NA	-
**mRS at last follow-up**	0	12 (85.7)	9 (36)	** < 0.01**
	1	1 (7.1)	7 (28)	0.21
	2	1 (7.1)	3 (12)	1.00
	3	0 (0)	0 (0)	1.00
	4	0 (0)	1 (4)	1.00
	5	0 (0)	0 (0)	1.00
	6	0 (0)	5 (20)	0.13
**Mean clinical follow-up (months)**		31.1 ± 19.9	39.2 ± 18.9	0.21
**Time btw index and bystanders treatment (months)**		14.3 ± 19.2	NA	**-**
**Treatment-related complications**	**Ischemia**	0	NA	-
	**Intraoperative rupture**	0	NA	-
	**Postoperative hemorrhage**	0	NA	-
	**Parent artery occlusion**	0	NA	-

*Btw*, between; *mRS*, modified Rankin scale; *NA*, not applicable

As regards the treatment modalities, after the institutional neurovascular board consensus, about half of the bystanders underwent open surgery and half endovascular treatment. Overall, we observed the occurrence of minimal remnants (< 5% of the aneurysm volume) in 3 cases at the last neuroradiological follow-up.

No cases of aneurysm growth or bleeding from bystanders were observed within the two groups along the entire follow-up period, which was longer than 12 months for the treated group (time between the index and bystanders treatment: 14.3 ± 19.2 months), and almost 40 months for the patients who underwent observation (39.2 ± 18.9).

Finally, no major complications occurred during the bystanders treatment determining mRS score modifications compared to the preoperative quality of life.

## Discussion

In case of SAH associated with MIAs, indications and timing of the bystanders treatment are still debated.

Several authors reported as effective the simultaneous treatments of MIAs associated with SAH [2–8], but even when technically feasible, the benefit of a simultaneous treatment of index and bystanders remains questionable. In fact, whether on the one hand it appears convenient as patients are subjected to only one procedure to secure all aneurysms, on the other this could be not necessarily the best choice in terms of safety due to the difficult management of a hemorrhagic brain and the higher risk of vasospasm, which is entailed by both surgical and endovascular procedures.

### Risk of bystander aneurysm rupture

As regards patients with SAH associated with MIAs, three main issues were explored in literature:

(1) if their clinical outcome is different compared with patients presenting SAH associated with a single aneurysm; (2) which is the risk of SAH in patients harboring multiple unruptured aneurysms; (3) and which is the risk of bystander rupture after a previous SAH from another aneurysm.

(1) As regards the first question, some authors reported that the presence of MIAs is associated with a higher rate of post-treatment stroke and new focal neurological deficits compared with patients suffering from SAH associated with a single aneurysm; however, no significant differences in overall functional status and survival were observed [9, 10]. Conversely, other studies did not find any association between the detection of MIAs at the time of the SAH and a worse outcome after treatment [11, 12]. Thus, the real influence of the presence of MIAs on the outcome after SAH is not yet established.

(2) As regards the second question, harboring MIAs has been reported as a risk factor for a higher risk of SAH by several authors [13–15] so the aneurysm multiplicity was included in the UIATS recommendation score as a variable supporting the indication to treat [16].

However, this association appears controversial as there are two interpretations of this increased risk. The first is that the same risk factors (female sex, smoking history, posterior circulation topography) influencing the incidence of MIAs also affect the risk of rupture [11]. The second, instead, simply considers the higher risk of bleeding just as a cumulative risk of multiple aneurysms in the same patient [17].

Contrariwise, some other studies did not find any association between MIAs and a higher risk of SAH [18, 19].

(3) Finally as regards the third question, it is widely believed that the risk of bystander rupture in patients with a previous SAH is higher compared with patients with MIAs without a history of SAH and patients with single aneurysms. In agreement, the occurrence of a previous SAH was included among the risk variables of PHASES score [18] and UIATS recommendation scores [16]. However, this association was not confirmed by all studies, in particular when confounding factors affecting both aneurysm rupture and multiple aneurysm occurrence were considered [20].

### Risk of bystander aneurysm growth

With regard to the risk of aneurysm growth over time, which in turn is commonly considered relevant for future rupture, it was observed to be only marginally influenced from the occurrence of a previous SAH in the same patient. Two meta-analyses, in fact, largely investigated the factors associated with aneurysm growth, but they did not find a significant influence of a previous SAH on the risk of bystanders morphological changes [21–23].

Moreover, in the original ELAPSS study, which was conceived to measure the risk of growth of unruptured aneurysms over time in order to help draw recommendations for neuroradiological follow-up, a previous SAH was found even negatively scoring on the risk of aneurysm growth [24]. However, ELAPSS did not include as primary endpoint measuring the risk of aneurysm bleeding and did not directly explored the role of a previous SAH on the risk of aneurysm rupture as it has been done by PHASES and UIATS score systems.

Therefore, as aneurysm growth was largely demonstrated directly influencing a future risk of rupture, this evidence appears somewhat contradictory.

### Timing of bystander aneurysm treatment

In this controversial scenario, the choice to treat or not all aneurysms at the same time remains challenging.

Due to the absence of specific indications about the timing of bystanders treatment, the decision-making process is usually based on several clinical and neuroradiological considerations as well as the availability of expert interventional neuroradiologists and cerebrovascular surgeons.

Among the motivations supporting the indication for a concomitant treatment, there are both technical and clinical reasons. For instance, an ipsilateral topography to the index aneurysm in case of open surgery or being susceptible for a simple coiling stand-alone in the same endovascular session to treat the index aneurysm usually represents the principal technical reasons fostering a multiple aneurysm management at the same time after a SAH. Instead, particularly at risk angioarchitectural characteristics, some major patient risk factors such as ethnics, age, sex, previous SAH, hypertension, smoke, and, less often, the need for a prolonged antiplatelet/anticoagulants therapy are the main clinical reasons supporting to secure multiple aneurysms in the acute phase [17–20, 25–27].

Above all, however, this choice is affected by the clinical status of the patient after the SAH, since in the case of more severe conditions, the general tendency of all neurosurgeons is to limit the procedural risks in the acute phase, securing only the index aneurysm, and postpone the bystanders treatment to an elective setting limitedly to the patients showing a functional recovery.

### Finding of the present study

In our experience, except for cases of MIAs in which we were not able to demonstrate with certainty the source of the bleeding, the aim of the immediate treatment after a SAH was to exclusively secure the index aneurysm. Then, we usually discussed all cases harboring bystanders at the institutional neurovascular board to reach an agreement about a treatment proposal or just observation.

Patients addressed for treatment were usually younger and female, with good clinical recovery after the first SAH. The possibility of an endovascular coiling was instead the most common reason for offering treatment even to older patients.

However, when we retrospectively applied the PHASES score, we did not observe a significantly higher risk of rupture in the intervention group (Table 2) [16, 28–31]. Bystander aneurysms undergoing treatment were also significantly larger, with a higher AR and, more often, irregular morphology compared with the observation group.

Bystanders treatment was also effective and the occurrence of a previous SAH did not appear a limitation neither from a surgical nor from an endovascular point of view. Only three minimal remnants were observed as a result of a minimal neck sparing to guarantee the collateral perfusion in two cases of PICA and PCom aneurysms with junctional morphology [32], and another due to a still incomplete obliteration in a flow-diverted ophthalmic ICA aneurysm.

Along the entire follow-up period, no cases of SAH from bystander rupture were observed neither in the treated group (mean follow-up 14.3 ± 19.2 months) nor in the observation group (mean follow-up 39.2 ± 18.9).

The range of time between treatments of index and bystander aneurysms varied according to three main parameters: post-SAH neurological recovery, angioarchitectural characteristics of the aneurysms, and patients’ preference.

### *Limitations*

This is a single-center study with a retrospective setting and including a limited number of patients. Moreover, we cannot exclude a certain selection bias due to the fact that the indication to treat or to observe was based on an institutional guideline. Finally, we could explore only a limited follow-up period of about 40 months for the conservative group, which is however only partially generalizable to a life-long risk.

Despite the presence of these limitations, our experience does not support a mandatory need for a contemporary treatment of index and bystanders.

On the other hand, for bystanders considered having a not negligible risk of rupture, the treatment at a distance from a previous SAH can be considered as it seems having a risk profile comparable with the treatment of unruptured aneurysms in patients with no previous history of SAH. This is supported by the fact that no clinical worsening was observed in our patients after the treatment of a bystander.

## Conclusions

In case of SAH associated with MIAs, the aim of the acute treatment is identifying and securing the ruptured aneurysm. The treatment of the bystanders, especially when topographically distant from the index aneurysm, can be postponed to an elective setup, after that the patient recovered a good clinical condition as within the short follow-up, intervention and observation seem to be likewise safe for aneurysms. On the other hand, the risk of delayed treatment of these aneurysms does not seem to be different from the treatment of unruptured aneurysms in patients without a previous history of SAH.

## Data Availability

Available upon reasonable request.

## Abbreviations

SAH: subarachnoid hemorrhage
MIAs: multiple intracranial aneurysms
mRS: modified Rankin Scale
CTA: CT-angiography
MRA: magnetic resonance angiography
DSA: digital subtraction angiography
HH: Hunt-Hess
